# Spatial effects of public health laboratory emergency testing institutions under COVID-19 in China

**DOI:** 10.1186/s12939-023-01871-0

**Published:** 2023-05-15

**Authors:** Baoguo Shi, Yanjie Wang, Xiaodan Bai, Yongqiang Lai, Wenjing Xiang, Bing Wu, Qi Xia, Xinwei Liu, Ye Li

**Affiliations:** 1 Department of Economics, School of Economics, Minzu University of China, Beijing, China; 2 Research Center for Health Policy and Management, School of Health Management, Harbin Medical University, Harbin, Heilongjiang, 150086 China

**Keywords:** COVID-19, Nucleic acid testing (NAT) institutions, Spatial heterogeneity, MGWR-SAR method

## Abstract

**Background:**

The transmission of 2019 novel coronavirus (COVID-19) has caused global panic in the past three years. Countries have learned an important lesson in the practice of responding to COVID-19 pandemic: timely and accurate diagnosis is critical. As an important technology of virus diagnosis, nucleic acid testing (NAT) is also widely used in the identification of other infectious diseases. However, geographic factors often constrain the provision of public health services such as NAT services, and the spatial nature of their resource allocation is a significant problem.

**Methods:**

We used OLS, OLS-SAR, GWR, GWR-SAR, MGWR, and MGWR-SAR models to identify the determinants of spatial difference and spatial heterogeneity affecting NAT institutions in China.

**Results:**

Firstly, we identify that the distribution of NAT institutions in China shows a clear spatial agglomeration, with an overall trend of increasing distribution from west to east. There is significant spatial heterogeneity in Chinese NAT institutions. Secondly, the MGWR-SAR model results show that city level, population density, number of tertiary hospitals and number of public health emergency outbreaks are important factors influencing the spatial heterogeneity of NAT institutions in China.

**Conclusions:**

Therefore, the government should allocate health resources rationally, optimise the spatial layout of testing facilities, and improve the ability to respond to public health emergencies. Meanwhile, third-party testing facilities need to focus on their role in the public health emergency response system as a market force to alleviate the inequitable allocation of health resources between regions. By taking these measures to prepare adequately for possible future public health emergencies.

## Background

Epidemic prevention and control all start with diagnosis, which is the basis for successful outbreak control [1]. A strong public health emergency system requires the multidimensional support of diagnostic safety, vaccine safety, and treatment safety [2]. Among them is diagnostic safety, a benchmark element for the proper functioning of the health system, which is critical for a timely response to an outbreak [3]. Testing problems such as insufficient testing capacity and delayed test reports have hampered outbreak awareness and response, leading to a rapid influx of unreliable, inaccurate, and even erroneous tests, allowing rapid spread of the virus in a short period of time and increasing mortality and morbidity rates, jeopardizing global public health safety. In the initial outbreak of Ebola in West Africa in 2014, the disease was misdiagnosed as cholera and salad fever and was not officially diagnosed as Ebola until three months later when it was sent to a testing facility in Europe for testing [4]. When the yellow fever outbreak occurred in 2016, the relevant agencies were late in receiving laboratory diagnostic results, and the lack of testing capacity greatly reduced the effectiveness of outbreak control [5]. Other infectious diseases, such as Lassa fever, Middle East respiratory syndrome, ZIKA, and COVID-19 pandemic that is now prevalent around the world, have all experienced delays in diagnosis due to the lack of virus detection by testing institutions, which further affects the subsequent series of treatment and prevention and control [6–9]. The lesson to be learned from these experiences is that timely and accurate diagnosis is critical to the prevention and control of outbreaks [10, 11].

In addition, because of the immediate nature of public health emergencies, test results need to be reported in the shortest possible time, and the timeliness of disease response and the number of people living in the area need to be taken into account, so the testing agencies in the geographical distribution of fairness is particularly important, which is the first step in the process of public health emergencies in response to achieve rapid testing [12]. However, studies have shown that current access to virus testing is still unequal in different regions of the world [13]. A meeting report by World Health Organization suggests that access to NAT shows inequity as the economic gap develops [14]. During the pandemic of COVID-19, the number of reported cases of the COVID-19 pandemic was much lower than the actual number of cases in most African countries, due to inadequate health systems, insufficient number of relevant medical personnel, insufficient number of NAT facilities, and poor NAT capacity [15, 16]. When testing facilities are poorly accessible geographically, patients are not diagnosed in a timely manner and the quality of testing decreases, which can delay the outbreak and lead to more serious consequences [17–20].

Previous research on detection of outbreaks of infectious diseases have focused on the technology [21–23] and methods [24–29] of viral testing itself, with less attention to issues of geographical accessibility and fairness of testing. However, inadequate detection systems and unequal access to reliable diagnostic tests often result in significant delays in all aspects of outbreak response, from initial detection to ultimate containment [30].

After the devastation of SARS in 2003, China began to build a public health emergency response system based on the established public health system to prepare technical reserves for virus detection and diagnosis in the early stages of major outbreaks [31]. China has issued a series of national policy documents, gradually improving many aspects such as laboratory construction, virus detection technology, testing personnel, testing and reporting mechanisms, and coordination mechanisms with external organizations, etc., to integrate the construction of public health should system into daily life [32–34]. China relied mainly on NAT techniques for virus identification during the COVID-19epidemic [35]. There are three types of healthcare institutions that provide NAT services in China, namely medical institutions, CDC institutions and third-party medical testing laboratories [36]. Among them, medical institutions are the main aspect of nucleic acid testing work, accounting for the largest share of the total number of NAT institutions. There are serious inequalities in the current geographic distribution of high-quality medical resources, and the geographic spatial configuration characteristics of NAT institutions that depend on the routine laboratory construction situation of high-quality medical resources also deserve attention [37]. This leads us to ask.

What factors influence the setup of NAT institutions in China? Will the number of NAT institutions set up in one region be influenced by neighboring regions? How does the number of NAT institutions in a region vary by area, population, and morbidity? Is the spatial distribution of NAT institutions characterized by both heterogeneity across regions and autocorrelation with neighboring regions? Addressing these issues is an assessment of China's emergency response capacity in the face of major public health events and an exploration of the equity of resource allocation across China's vast regions. They require cutting-edge research models as tools to accurately identify the spatial distribution characteristics of NAT institutions by comparatively assessing the model fitness of different functions [38–41].

Taking NAT institutions as an example, this aritle depicts the spatial pattern of NAT institutions in China and discusses their influencing factors, aiming to provide scientific references for further development of the spatial allocation of laboratory emergency testing institution resources under public health emergencies in China and other countries around the world.

## Methods and models

### Data sources

This paper takes the NAT institutions of 364 prefectural administrative units in China as the research object (Excluding the data of Hong Kong Special Administrative Region and Taiwan Region). The data of China's NAT institutions are obtained from the National Government Service Platform. At present, more than 9,700 NAT institutions have been set up in China, with an average of 315 testing institutions per province, region or municipality directly under the central government, providing security for virus screening of people and goods everywhere. Of these, 7,211 are in the hospital category, accounting for 73.8% of the total; more than 1,900 are in the CDC category, almost one-fifth of the total; and more than 600 are third-party NAT institutions. By downloading the latitude and longitude of each NAT institutions, using ARCGIS10.2 and other software, the geographic coordinates of the NAT institutions are matched with the research regions above.

The factors influencing the layout of NAT institutions include epidemiological factors, economic and social factors, political factors, historical factors (and physical geographical factors. The epidemic factors include the number of epidemic outbreaks and the cumulative case numbers of new coronary pneumonia infections, with data obtained from publicly available data on the Chinese government website. The indicators that characterise the level of economic development include Population density, GDP per capita, urbanisation rate and city level, which comes from the "2019 Statistical Bulletin of Economic and Social Development" of each prefecture-level unit. The indicators of historical factors mainly include medical education, that is, the number of medical universities or medical colleges, the data of which comes from the directory of China Medical University (Medical College) and the affiliated hospitals. The data is obtained from the list of municipalities directly under the Central Government, provincial capital cities, and cities under separate planning. Please refer to Table 1 for the definition and source of all the above variables.

**Table 1:** Description of variable

Category	Variable	Definition	Unit	Mean	SD	Min	Max	Vif
Dependent Variable	Number of testing institutions	Number of testing institutions in an administrative area	N/A	26.783	27.971	1	270	—
Independent Variables	City level	All cities are divided into two criteria: value = 1: Prefecture-level area value = 2: City specifically designated in the state plan or municipality directly under the Central Government	N/A	0.082	0.275	0	1	2.423510
	GDP per capital	Gross Domestic Product per capital	Yuan per capital	54,115.03	32,642.09	4742	183,127	2.009221
	Population density	Population per unit land area	Person per square kilometer	419.236	553.044	1	6111	1.572160
	Urbanisation rate	Urban population/ Permanent population	%	54.468	14.931	5	100	2.051932
	Altitude	The vertical distance of a point on the ground above sea level	Mile	564.321	825.298	1	4507	1.292968
	Number of medical colleges	Number of medical colleges in an administrative area	N/A	0.233	0.750	0	6	4.012514
	casecount	Cumulative number of confirmed cases in administrative area	N/A	246.601	2667.228	0	50,380	1.088747
	outbreaks	Number of outbreaks in the administrative area	N/A	1.119	0.662	0	5	1.403457
	Number of tertiary hospitals	Number of tertiary hospitals in an administrative area	N/A	3.155	4.916	0	42	5.590554

### Exploratory Spatial Data Analysis (ESDA)

Our research used a six-model residual spatial autocorrelation approach to explore the mitigating effect of the inclusion of a lag term in analysing of the spatial agglomeration of NAT institutions in China.

Generally, Moran's I is introduced to measure the global spatial correlation features. This is a method used for global clustering tests in the entire study area that is similar and different (spatial positive correlation, negative correlation) or independent of each other. Global spatial autocorrelation generally uses Moran's I index [42, 43]. Moran's I index is between − 1 and 1, and its calculation formula is as follows:

1 $$I=\frac{n\sum_i\sum_jW_{ij}(X_i-\overline X)(X_j-\overline X)}{(\sum_i\sum_jW_{ij})\sum_i(X_i-\overline X)^2}$$

In the formula: n is the total number of areas in the study, ${\rm X}_{i}$ and ${\rm X}_{j}$ are the numbers of NAT institutions in areas in i and j;${W}_{ij}$ are the spatial weight matrix, spatial adjacent is 1 and non-adjacent is 0; $\overline{\mathrm{X} }$ is the average value of numbers of NAT institutions. Perform statistical tests on Moran's I results, usually using Z-test [43]:

2 $$\mathrm Z(\mathrm I)=\frac{\text{Moran's I}-\mathrm E(\mathrm I)}{\sqrt{\mathrm{VAR}(\mathrm I)}}$$

E(I) is the mathematical expectation, var (I) is the variance.

### Econometric framework

#### Ordinary Least Squares (OLS)

Ordinary least squares (OLS) seeks to minimise the sum of the squares of the distances from all observations in the scatter plot to the regression line, which is the most fundamental form of regression analysis, therefore, the first method must be used in regression analysis. The calculation formula is as follows: 

3 $$y_{i\;}=\beta_0+{\textstyle\sum_{k\;}}\beta_k\;x_{ik}+\;\varepsilon_i\\$$

Where i denotes the prefecture-level city number,${y}_{i}$ denotes the number of NAT institutions in city i,${x}_{ik}$ denotes the kth covariate for city i, in this paper k takes all integers in the interval [24, 42], denoting respectively: urbanisation rate, GDP per capita, altitude, number of medical colleges, city level, population density, number of tertiary hospitals, casecount and outbreaks.${\beta }_{0}$ is the expected value of the number of NAT institutions in each region when all explanatory variables do not play a role, ${\beta }_{k}$ is the kth regression parameter of the covariate, which indicates the fluctuation of ${y}_{i}$ as the covariate ${x}_{ik}$ changes, ${\varepsilon }_{i}$ is the random error term.

#### Geographically Weighted Regression(GWR)

This method is based on the local regression analysis method, incorporates the data's spatial location into the regression parameters, and uses the local weighted least square method to estimate point-by-point parameters [44, 45]. The estimated parameters of each spatial unit change with geographic spatial location, thereby directly displaying the spatial heterogeneity of the research object in the research area. Geographically weighted regression can also be regarded as an extension of the traditional global regression model. Its formula is

4 $${y}_{i}={\beta }_{0}({u}_{i},{v}_{i})+{\sum }_{k}{\beta }_{k}({u}_{i},{v}_{i}){x}_{ik}+{\varepsilon }_{i}$$

where i is the prefecture-level city number, ${y}_{i}$ is the number of NAT institutions in city i, ${x}_{ik}$ is the kth covariate for city i, $({u}_{i},{v}_{i})$ is the centre-of-mass coordinates for city i, ${\beta }_{k}\left({u}_{i},{v}_{i}\right)$ is the kth regression parameter for city i, which measures the effect of changes in different covariates on the dependent variable, ${\beta }_{0}({u}_{i},{v}_{i})$ is a constant term, ${\varepsilon }_{i}$ is a random error term.

Meanwhile, this paper chooses the adaptive bandwidth quadratic kernel function commonly used in academia as the distance weighting function, i.e.

5 $${\mathrm w}_{\mathrm{ij}}=\left\{\begin{array}{cc}\left[1-\left(\frac{{\mathrm d}_{\mathrm{ij}}}{\mathrm b}\right)^2\right]^2&\mathrm{if}\;{\mathrm d}_{\mathrm{ij}}<\mathrm b\\0&\mathrm{otherwise}\end{array}\right.$$

and b is the distance between spatial units from regression position i to its kth nearest.where $w{\text{ij}}$ is the weight between city i and city j,$dij$ is the distance between city i and city j, b is a critical distance from regression location i to its k-th nearest neighbor.

#### Multi-scale Geographically Weighted Regression (MGWR)

The estimated bandwidth (the number of prefecture-level units used for local estimation) of each relationship in GWR is the same, which has some limitations. The recently developed multi-scale geographically weighted regression (MGWR) improves the GWR, which relaxes the assumption of "same spatial scale" and optimises covariate specific bandwidth [44]. Its formula is:

6 $${y}_{i}={\beta }_{h0}({u}_{i},{v}_{i})+{\sum }_{k}{\beta }_{hk}({u}_{i},{v}_{i}){x}_{ik}+{\varepsilon }_{i}$$

where i denotes the prefecture-level city number, h denotes the specific optimal bandwidth used to calibrate the different conditional relationships, ${y}_{i}$ denotes the number of NAT institutions in city i,${x}_{ik}$ denotes the kth covariate for the ith city, $({u}_{i},{v}_{i})$ denotes the centre − of − mass coordinates of city i,${\beta }_{hk}({u}_{i},{v}_{i})$ is the kth regression parameter for city i at the specific optimal bandwidth h, measures the extent to which fluctuations in different covariates affect the number of NAT institutions, ${\beta }_{h0}({u}_{i},{v}_{i})$ is a constant term, ${\varepsilon }_{i}$ is the random error term.

#### GWR-SAR and MGWR-SAR

We create a separate GWR Model and MGWR model by adding the spatial lag term to the covariate and combining the model's multi-scale GWR term with the lag dependent variable (MGWR-SAR) [46]. The generated spatial weight matrix W is multiplied by the column vector represented by the dependent variable Y. By lagging Y once, the resulting column vector WY is added as a new variable to the covariates, and then GWR and MGWR regressions are performed. The GWR-SAR and MGWR-SAR models can precisely portray the two effects of spatial autocorrelation and spatial heterogeneity at the same time [39, 40].

#### GWR-SAR

The autocorrelation results of the dependent variable were added as independent variables on the basis of the GWR model to portray the spatial autocorrelation of the NAT institutions. The specific formula is shown below [40].

7 $${\mathrm y}_{\mathrm i}={\mathrm\beta}_0({\mathrm u}_{\mathrm i},\;{\mathrm v}_{\mathrm i})+{\mathrm\beta}_1({\mathrm u}_{\mathrm i},\;{\mathrm v}_{\mathrm i})W_y+\sum_{\mathrm k=2}\;{\mathrm\beta}_{\mathrm k}({\mathrm u}_{\mathrm i},\;{\mathrm v}_{\mathrm i}){\mathrm x}_{\mathrm{ik}}+{\mathrm\varepsilon}_{\mathrm i}$$

The symbols here represent the same meaning as in the GWR formula. ${\upbeta }_{1}$ is the spatial autoregressive parameter, W represents a $\mathrm{n}\times \mathrm{n}$ spatial weight matrix.

#### MGWR-SAR

A limitation of GWR is that the estimated bandwidth is the same for each relationship in the model. MGWR can provide covariate specific optimization bandwidths. Like GWR-SAR, MGWR-SAR can also portray both spatial autocorrelation and spatial heterogeneity. Its expression is shown below [38].

8 $$y_i=\beta_{h0\;}(u_i\;,\;v_i)\;+\;\beta_{h1}(u_i,\;v_i)\;w_y\;+\sum_{k=2}\;\;\beta_{hk}\;(u_i,\;v_i)\;x_{ik}\;+\;\varepsilon_i$$

The symbols here represent the same meaning as in the MGWR formula. ${\upbeta }_{\mathrm{h}1}$ is the spatial autoregressive parameter, W represents a $\mathrm{n}\times \mathrm{n}$ spatial weight matrix.

**Fig. 1:**
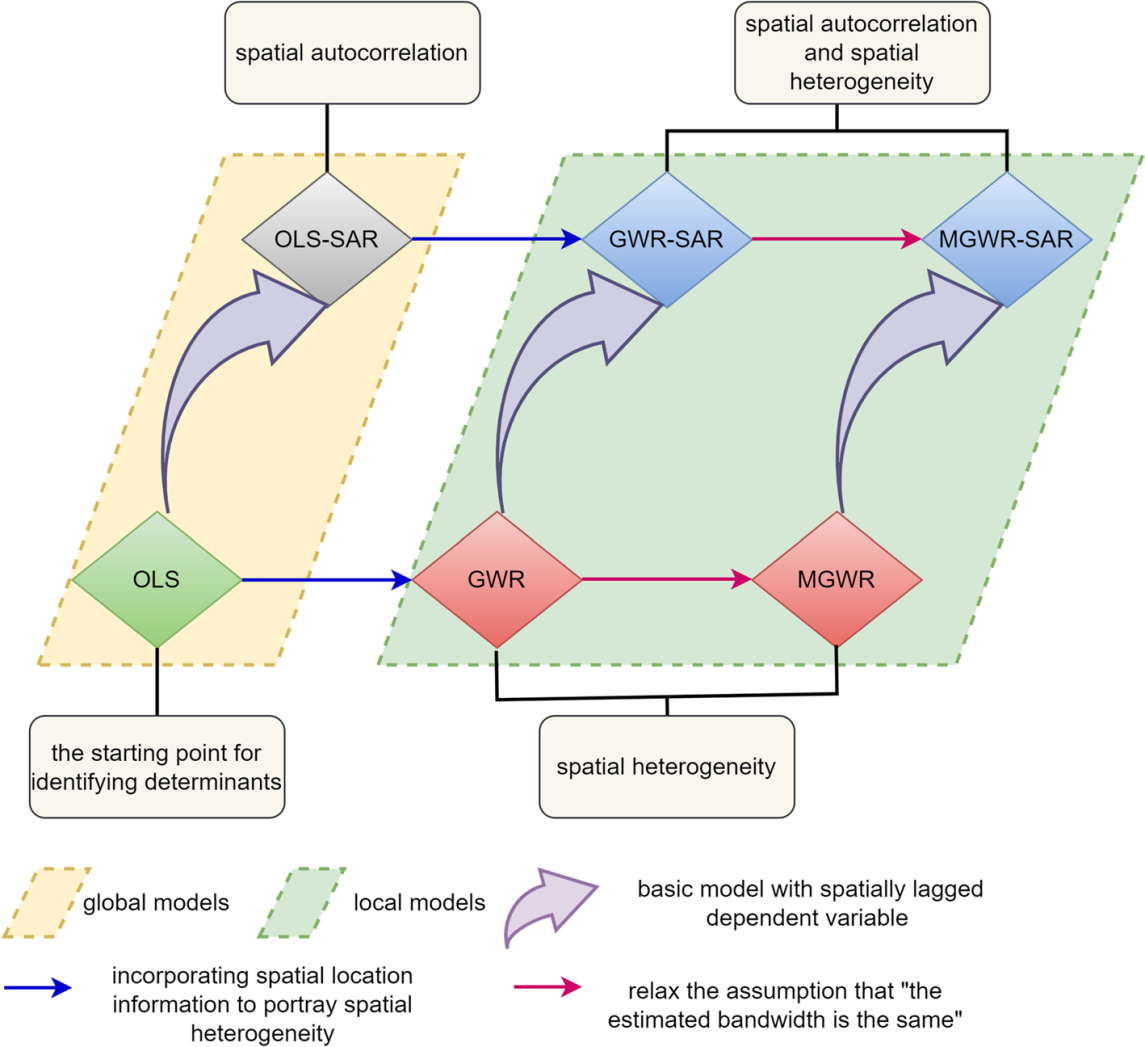
Methodology flowchart

Figure 1 shows the methodology flowchart we drew. We do not know whether there are heterogeneity and autocorrelation in the spatial patterns of NAT institutions. Therefore, we need to determine the spatial characteristics by the fit these six models and select the optimal one to accurately and specifically portray the spatial characteristics of the NAT institutions. We used MGWR2.2 software for all calibrations (https://sgsup.ASU.edu/SPARC/mgwr).

## Result

### Spatial configuration analysis of NAT institutions in China

**Fig. 2:**
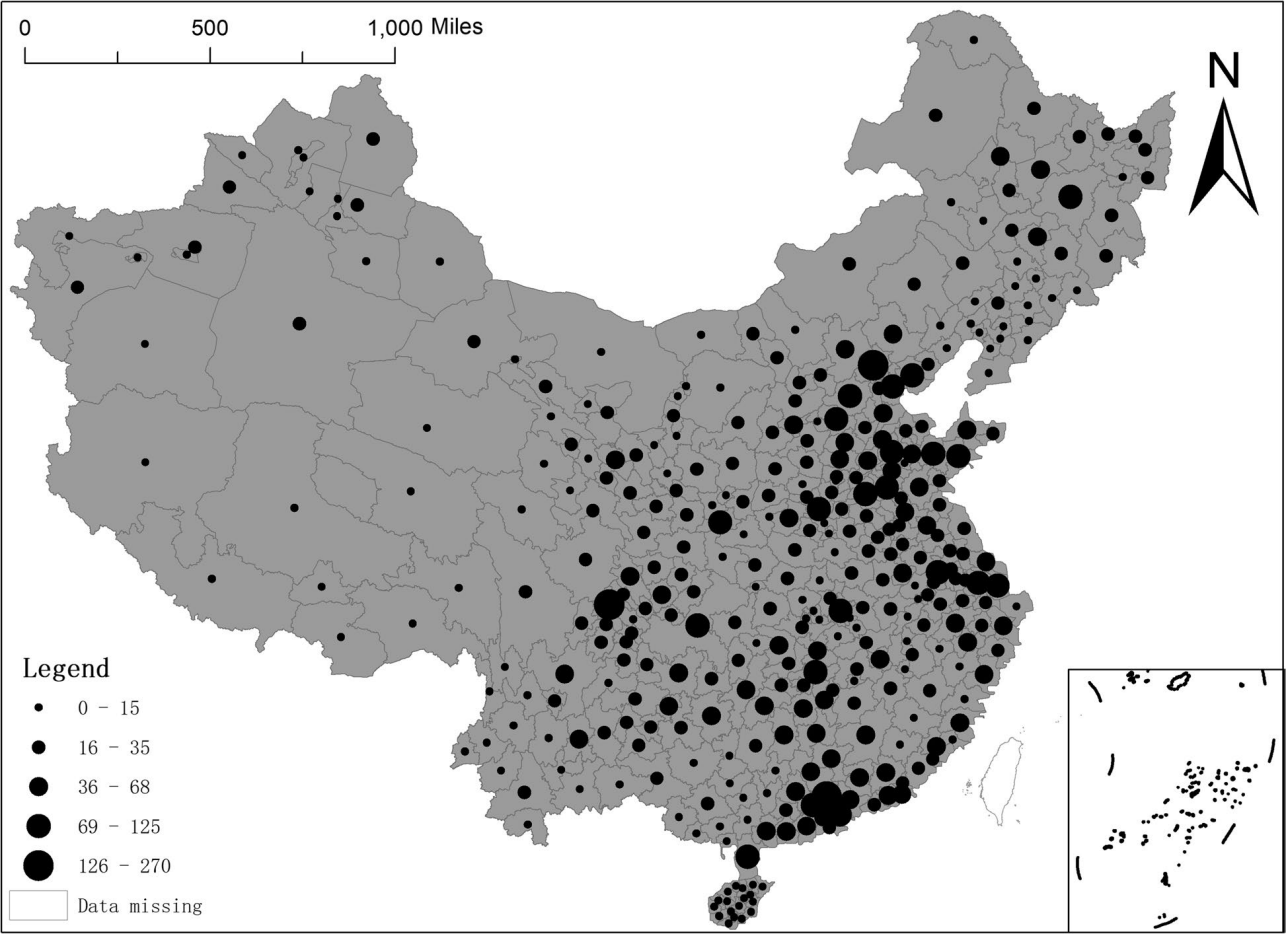
Spatial distribution of NAT institutions in China

The spatial distribution of NAT institutions in China is shown in Fig. 2. At the prefectural- level, 10 cities have more than 100 NAT institutions. The city with the highest number of NAT institutions is Beijing with 270, followed respectively by Chengdu (176), Guangzhou (169), Shenzhen (125), Shanghai (108), Baoding (106), Zhanjiang (106), Chongqing (103), Xi'an (102) and Foshan (102). 354 prefecture-level units have one or more NAT institutions, accounting for 98.1% of the total observations in all prefecture-level cities. At the provincial level, there are 12 provinces (regions and municipalities) with more than 300 NAT institutions, with Guangdong having the most (1289), followed by Shandong (866), Sichuan (699), Hebei (615), Hunan (560), Jiangsu (483), Anhui (408), Henan (396), Heilongjiang (391), Zhejiang (364), Hubei (313) and Gansu (304). The Tibet Autonomous Region has the lowest number of NAT institutions, with only 17. There are 4,345 NAT institutions at the regional level in 10 provinces in the eastern region, 2,173 in 6 provinces in the central region, 2,537 in 12 provinces in the western region, and 694 in the three northeastern provinces. As shown from Fig. 2, there is a clear concentration of NAT institutions in the eastern and southeastern coastal regions. At the same time, municipalities and densely populated regions also have a relatively higher number of testing institutions, such as Shandong, Guangdong, Chongqing and Beijing. In addition, the number of NAT institutions established in the Northeast and Western regions is more limited overall than in other regions. This study systematically and comprehensively analyses the possible influencing factors of the number of regional NAT institutions under the multiple dimensions of geography, economy, and society. Thus, we get the different effects of many factors on establishing NAT institutions and epidemic prevention and control. Meanwhile, theoretical support can be provided in public health security and long-term policy-making by our study.

### Analysis of factors influencing the space for NAT institutions in China

In order to avoid large deviations in the regression results due to the existence of collinearity between the explanatory variables, the variance inflation factor (VIF) was applied in this paper to test for collinearity in the explanatory variables (the results are shown in Table 1). The variance inflation factors (VIFs) of all the explanatory variables in the table are fairly low, all below 7.5, indicating that there is no collinearity between the variables.

Table 2 shows that the Moran I values are significantly smaller for the model with the lag term added compared to the original model, further indicating that the model with the lag term added emphasises the issue of spatial heterogeneity of Chinese NAT institutions more than the original model.

**Table 2:** Spatial autocorrelation of residuals

Model	Moran I	Z value	*P* value	Expected index
OLS	0.161713	11.921839	0.000000	-0.002755
OLS-SAR	0.098519	7.346418	0.000000	-0.002755
GWR	0.160338	11.799161	0.000000	-0.002755
GWR-SAR	0.080818	6.060106	0.000000	-0.002755
MGWR	0.164549	12.078296	0.000000	-0.002755
MGWR-SAR	0.068211	5.131994	0.000000	-0.002755

**Fig. 3:**
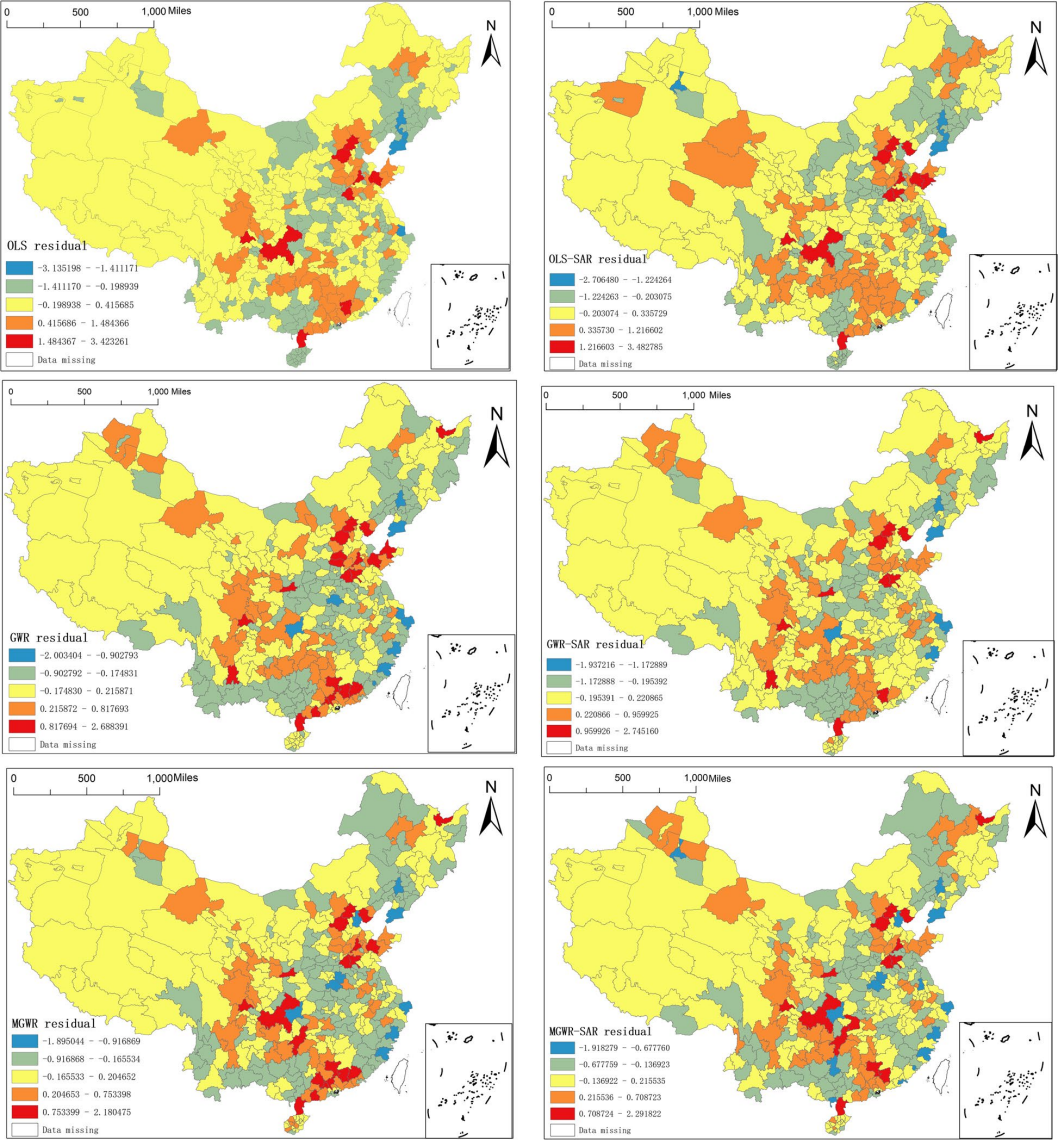
Spatial distribution of residuals for the six models

Figure 3 offers a more intuitive view of the spatial distribution patterns of the residuals generated by these six models. Where a, b, c, d, e, and f represents the spatial distribution patterns of residuals for the OLS model, the OLS-SAR model, the GWR model, the GWR-SAR model, the MGWR model, and the MGWR-SAR model, respectively. Figure 3 shows that the residual spatial agglomeration effect of the three models with the inclusion of the lag term, namely, the OLS-SAR. GWR-SAR, and MGWR-SAR models, is weaker but more suitable for studying spatial heterogeneity.

### Spatial Heterogeneity Analysis of NAT institutions in China Comparison of model fit superiority

In Table 3, four parameters, namely, Residual sum of squares, R^2^, Log-likelihood, and AICc value, are chosen to compare the six models in order to show the goodness of fit of various models more intuitively. First, when all the models add the spatial lag term, the goodness of fit of the models improves, that is, the OLS-SAR model fits better than the OLS model, the GWR-SAR model fits better than the GWR model, and the MGWR-SAR model fits better than the MGWR model because the Residual sum of squares and AICc value of the models become smaller and R^2^ becomes larger after adding the lag term. By comparison, we find that the Residual sum of squares of 61.222 and AICc value of 527.619 in the MGWR-SAR model are the smallest among all models, while its R^2^ was 0.f832, which was the largest among all models, indicating that the MGWR-SAR model had a better fit. In addition, Table 3 also shows the applicability of these six models in describing spatial features, among which the GWR-SAR and MGWR-SAR models are able to portray both spatial autocorrelation and spatial heterogeneity, the difference between them being that the former uses the same bandwidth and the latter uses a different bandwidth.

**Table 3:** Fitting of six models

Model	RSS	R^2^	Log likelihood	AICc value	Bandwidth	Spatial autocorrelation	Spatial heterogeneity
OLS	153.887	0.577	-359.803	742.356	---	---	---
OLS-SAR	141.265	0.612	-344.227	710.454	---	yes	---
GWR	90.013	0.753	-262.203	654.925	75	---	yes
GWR-SAR	79.493	0.782	-239.583	625.360	75	yes	yes
MGWR	70.882	0.805	-218.717	577.039	170.2^a^	---	yes
MGWR-SAR	61.222	0.832	-192.052	527.619	183.7^a^	yes	yes

^a^represents average value

Table 4 can both acquire a comprehensive understanding of the influences of various factors on the number of NAT institutions (local models generate individual parameter estimates for each variable at each location), and test the extent of the role of various influences on the number of NAT institutions in the presence of spatial autocorrelation features.

**Table 4:** Parameter Estimates for OLS and OLS-SAR NAT Institutions Regression

Variable	OLS	OLS-SAR
Intercept term	0.000	0.000
City level	-0.048	-0.035
GDP per capital	0.003	0.014
Population density	0.224^a^	0.171^a^
Urbanization rate	-0.031	-0.055
Number of medical colleges	0.190^a^	0.195^a^
Altitude	-0.062	-0.055
Casecount	-0.062^b^	-0.055
Outbreaks	0.068^b^	0.064
Number of tertiary hospitals	0.464^a^	0.478^a^
Lag		0.195^a^

^a^ represents the 5% level of significance and ^b^ represents the 10% level of significance

In the OLS model in Table 4, the coefficient estimates for the three variables of population density, number of medical schools, and number of tertiary hospitals are significant at the 5% level of significance, while the coefficient estimates for the number of confirmed cases and the number of outbreaks are significant at the 10% level of significance. Meanwhile, the coefficients of population density, number of medical colleges, number of tertiary hospitals, and number of outbreaks were all positive, meaning that when the population density of a region increases, the number of medical colleges increases, the number of tertiary hospitals increases, and the number of new outbreaks in the region increases, all have a significant positive effect on the number of NAT institutions in China; the regression coefficient of confirmed cases was negative. In addition, the regression coefficients of the variables city level, GDP per capita level, urbanisation rate, and altitude did not pass the significance test. In contrast, in the OLS-SAR model, population density, number of medical colleges, and number of tertiary hospitals remained significant at the 5% level of significance, but the significance of the number of confirmed cases and the number of outbreaks disappeared.

It is worth noting that the regression coefficients of population density in the OLS-SAR model became smaller compared to the OLS model, while the regression coefficients of the number of medical colleges and the number of tertiary hospitals became larger. In addition, the regression coefficient of the lag term in the OLS-SAR model is positive 0.195 and significant at the 5% level, indicating that the analysis of the spatial distribution dependence of NAT institutions in China cannot be handled by the OLS model alone, whereas the OLS-SAR model can show that the distribution of NAT institutions is spatially dependent, that is, the number of nucleic acid institutions testing in a prefecture-level unit and The OLS-SAR model shows that the distribution of NAT institutions is spatially dependent, that is the number of NAT institutions in a prefecture-level unit and the number of NAT institutions in surrounding cities are related.

Table 5 shows the percentage of cities that pass the 90% level of significance test for all coefficients (each city will get its own regression coefficient) when applying the MGWR-SAR model. The last two columns (+ (%) and—(%)) in Table 5 indicate the percentage of significant positive coefficients and significant negative coefficients, respectively. We grouped all explanatory variables into three categories: (1) at the 10% significance level, no regression coefficients significantly demonstrates that the variable has an effect on the spatial distribution of NAT institutions, including GDP per capita, urbanisation rate, and confirmed cases of new epidemic outbreaks, all of which have a *p*-value significance share of 0. (2) at the 10% significance level, more than half of the regression coefficients significantly demonstrates the effect of this variable on the spatial distribution of NAT institutions, including city level, population density, number of outbreaks, and number of tertiary hospitals. And the percentages of the significance of the *P* values of their regression coefficients are 85.440%, 78.022%, 99.451%, and 69.505%, respectively, with the percentages of positive values of the significant regression coefficients being 49.839%, 100%, 100%, and 95.257%, indicating that the significant effect of these variables (except city level) on the spatial distribution of NAT institutions tends to be more positive. (3) At the 10% significance level, a very small percentage of regression coefficients significantly prove that the variable has an effect on the spatial distribution of NAT institutions, including the number of medical colleges and altitude, and the significant percentages of the *P* values of their regression coefficients are 20.055% and 0.824%, respectively. 98.630% of the data in the significant regression coefficient of the number of medical colleges are positive, while the data in the significant regression coefficient of the altitude data are negative, indicating that there is an opposite effect of both on the spatial distribution of NAT institutions, but this effect is very small or even negligible. It is also worth mentioning that the bandwidth of the lag term Lag is 89.000, its mean value is 0.176, and the minimum and maximum values are -0.035 and 0.399, respectively, and 73.626% of its regression coefficients can explain its significance at 10% significance level, of which the percentage of positive values is 80.970%, which further illustrates the adoption of MGWR- SAR model is necessary for this paper.

**Table 5:** Parameter estimation of MGWR-SAR

		MGWR-SAR coefficient			Percentage of cities by significance (90% level)		
Variable	Bandwidth	Mean	Min	Max	*P* ≤ 0.1 (%)	+ (%)	− (%)
Intercept term	55.000	-0.020	-0.603	0.516	57.418	51.196	48.804
City level	85.000	0.047	-0.300	0.416	85.440	49.839	50.161
GDP per capital	277.000	-0.006	-0.075	0.032	0.000	0.000	0.000
Population density	45.000	0.654	-0.027	1.700	78.022	100.000	0.000
Urbanization rate	307.000	-0.022	-0.051	0.002	0.000	0.000	0.000
Number of medical colleges	72.000	0.085	-0.274	0.705	20.055	98.630	1.370
Altitude	363.000	-0.054	-0.073	-0.053	0.824	0.000	100.000
Casecount	277.000	-0.008	-0.149	0.017	0.000	0.000	0.000
Outbreaks	353.000	0.062	0.056	0.123	99.451	100.000	0.000
Number of tertiary hospitals	98.000	0.276	-0.051	0.561	69.505	95.257	4.743
Lag	89.000	0.176	-0.035	0.399	73.626	80.970	19.030

In order to demonstrate more clearly the influence of various elements on the spatial allocation of NAT institutions in China, we will elaborate through its spatial allocation characteristics map.

**Fig. 4:**
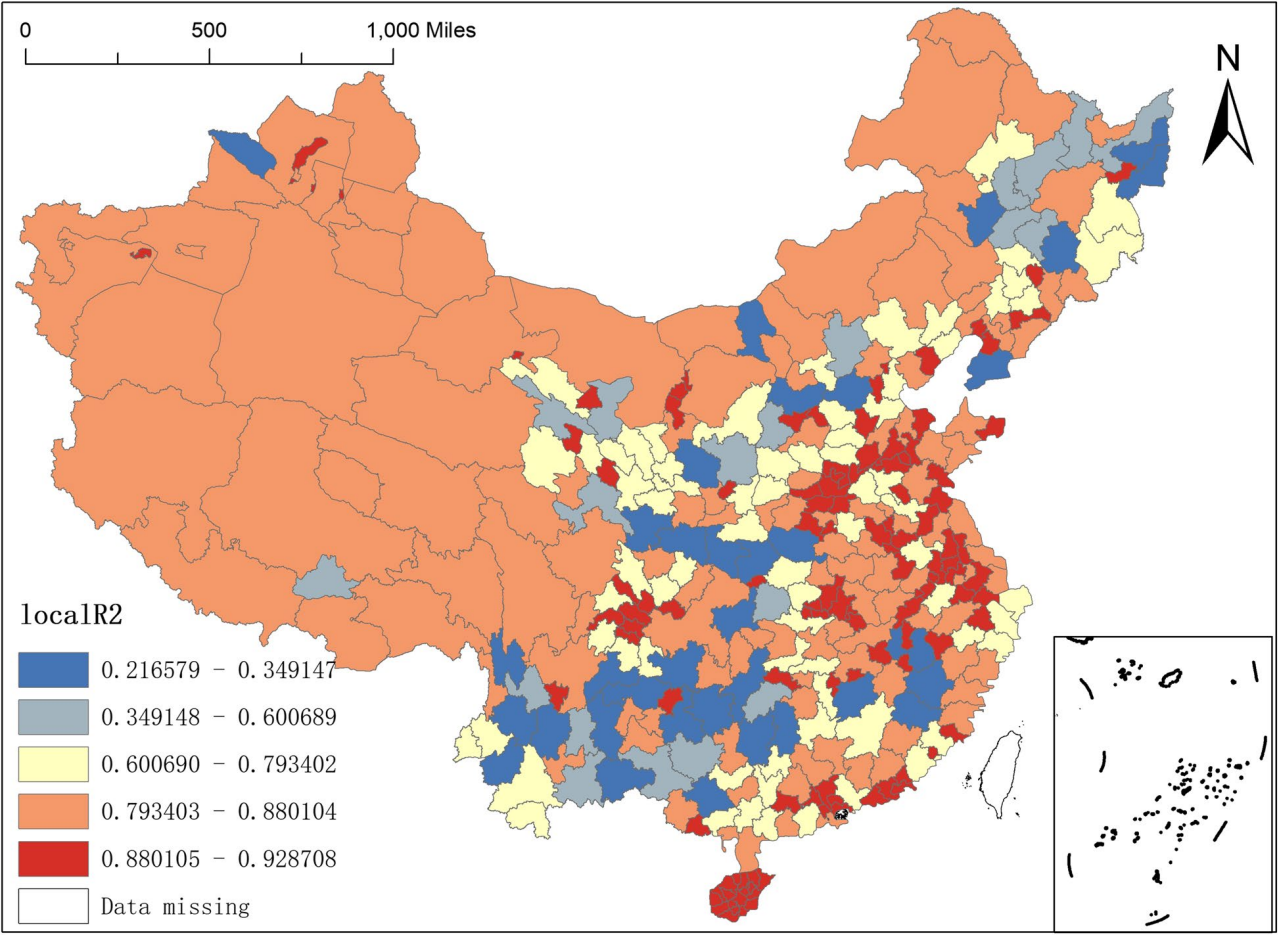
Spatial distribution of local R-squared of MGWR-SAR model

First, regarding the spatial differences in the local R^2^. The R^2^ of the MGWR-SAR model is 0.832, which is much higher than the R^2^ value of the OLS-SAR model of 0.612 and the R^2^ value of the MGWR model of 0.805. It is also higher than the R^2^ value of the GWR-SAR model of 0.782. Meanwhile, the RSS and AICc values of the MGWR-SAR model are the smallest among all models, indicating that the model is a better fit. This also illustrates that there is both spatial heterogeneity and spatial autocorrelation in the distribution of NAT institutions, and that the estimates are more accurate with different bandwidths. In Fig. 4, it can be seen that the minimum value of local R^2^ in the MGWR-SAR model is 0.2166 and the maximum value is 0.9287, and there are obvious spatial differences in local R^2^. The model is stronger in explaining the northwest as well as the central-east region, but weaker in explaining most of the cities in the southwest region. For this reason, according to the magnitude of the local R^2^, this paper divides China's prefecture-level units into five classes: (1) local R^2^ values between 0.2166 and 0.3495 are mainly distributed in Lincang, Dali, Chuxiong, Diqing, Wenshan, Qujing, and Bijie regions in the southwest, as well as in Boltara Autonomous Prefecture and Baotou in the northwest and cities such as Shuangyashan, Jixi, Baicheng, and Dalian in the northeast; (2) local R^2^ values between 0.3491 and 0.6007, mainly in Honghe Prefecture, Kunming and Lijiang in the southwest and Jiamusi, Yichun, Suihua, Daqing, Songyuan and Changchun in the northeast. (3) Local R^2^ values between 0.6007–0.7934, mainly distributed in some cities in the Middle East, including some cities in Zhejiang, Fujian, Guangdong, Hebei, Hunan and Hubei, in addition, Qiqihar, Mudanjiang, Tieling, Siping, Fushun, Shenyang in the Northeast and some provinces and cities in the western region, such as Qinghai and Shaanxi, are also in this range; (4) Local R^2^ values between 0.7934- 0.8801, this range of prefecture-level units covers a large area, almost the entire western region and is also widely distributed in Shandong, Jiangsu, Henan and Hubei in the central-eastern region; (5) local R^2^ values between 0.8801–0.9287, mainly distributed in the central-eastern region, including some cities in Hainan Province, Guangdong, Sichuan, Shandong, Henan and Anhui. Among them, the local R^2^ maximum value is in Guangzhou City, Guangdong Province.

**Fig. 5:**
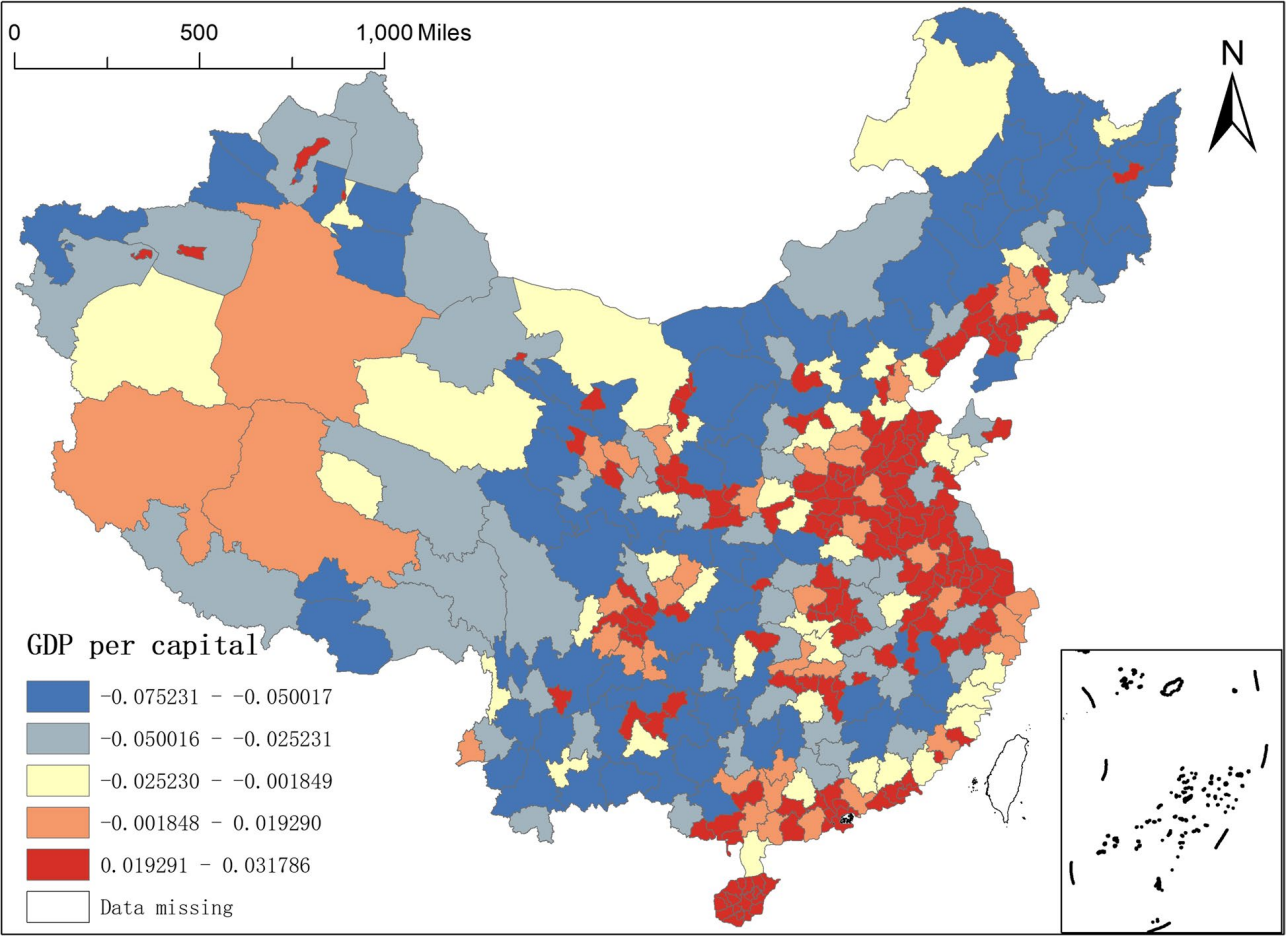
Spatial distribution of regression coefficients of GDP per capita

Second, the regression coefficients of GDP per capita and urbanisation rate. Both GDP per capita and urbanisation rate can be attributed to economic factors, where GDP per capita can measure the economic development of a region and is an effective instrument to understand and grasp the macroeconomic performance of a region. Figure 5 shows that the minimum value of the regression coefficient of GDP per capita is -0.0752 and the maximum value is 0.0318. It can also be seen that the coefficient of influence of the size of GDP per capita on the distribution of NAT institutions is large and positive in the southern part of South China and the central-eastern regions of Shandong, Anhui, Jiangsu, Hubei, and Liaoning, but some northeastern regions, northern China, southwestern regions, and northern Xinjiang Changji, Yili, Kizilsu, Karamay, Turpan, Bortala Prefecture, and Qinghai have smaller but negative coefficients of the effect of GDP size per capita on the distribution of NAT institutions, indicating that the explanatory strength of the regression coefficients of GDP per capita is relatively large in both parts, but with opposite effects.

**Fig. 6:**
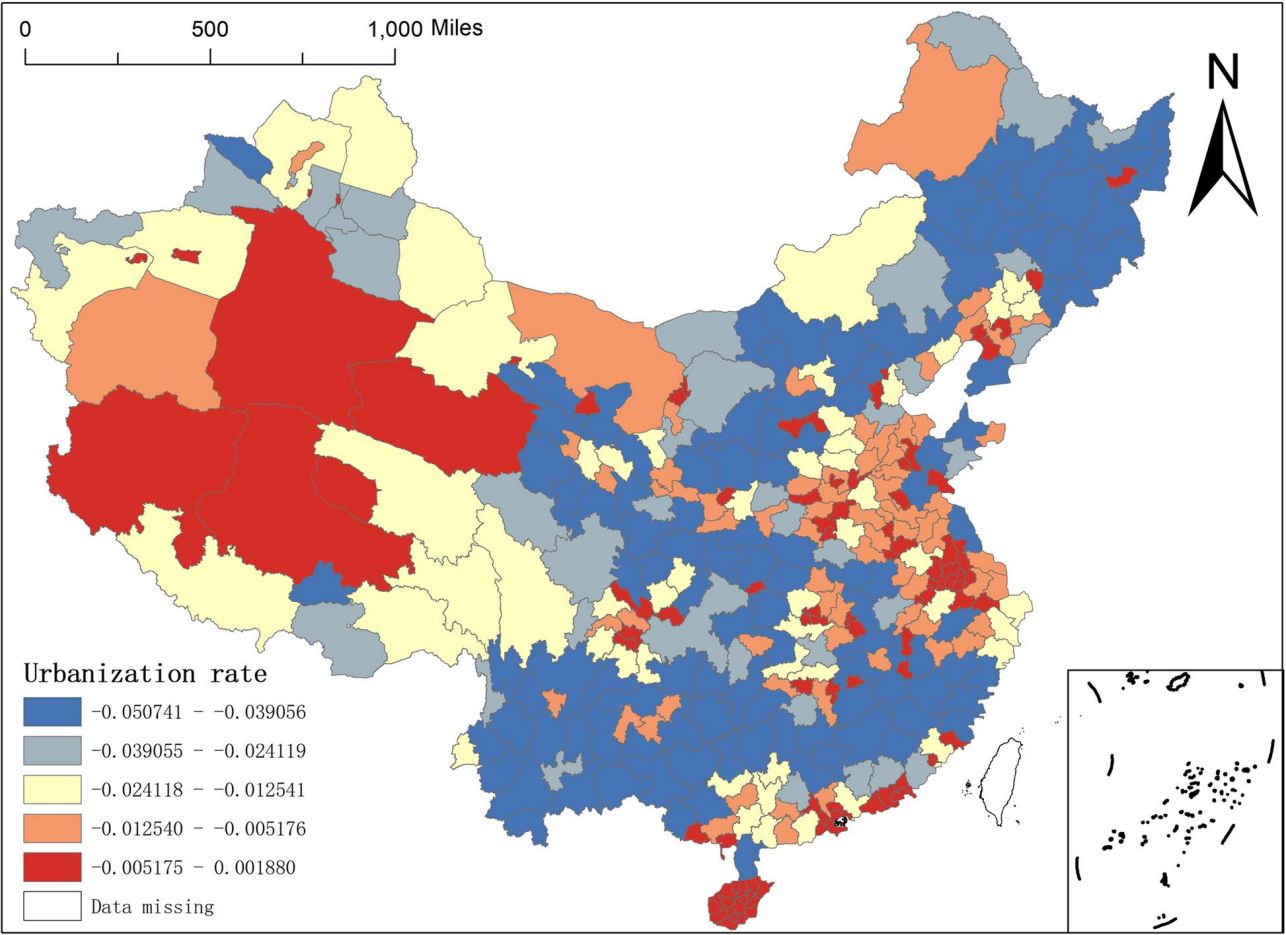
Spatial distribution of regression coefficients of urbanisation rate

On the other hand, the urbanisation rate is a sign of economic development of a country or region, and also an important indicator of the degree of social organisation and management of a country or region. Therefore, in this paper, the urbanisation rate rather than the urban population is included in the analysis framework, and Fig. 6 shows the spatial distribution of the regression coefficients of the urbanisation rate of the MGWR-SAR model, from which it can be found that: the regression coefficients of the urbanisation rate are overwhelmingly negative, and the maximum value is This indicates that the urbanisation rate has a negative impact on the distribution of the number of NAT institutions in China. The regions with larger regression coefficients are mainly distributed in Bayingoleng Mongol Autonomous Prefecture in Xinjiang, Ali and Nagqu regions in Tibet, and Hainan Province, and in some cities in the central and eastern part of China in a scattered form; while the regions with smaller regression coefficients (negative values) are mainly distributed in the northeast, south-central and western parts of Qinghai, Gansu and Inner Mongolia, indicating that in this geographical area, the regression coefficient of urbanisation rate has a strong negative effect on the spatial distribution of elite hospitals has a strong negative effect.

In summary, the absolute values of the regression coefficients of GDP per capita and urbanisation rate among the economic factors are low. And the distribution of NAT institutions in China is related to them to some extent, but not deeply, suggesting that there are other factors that will have a more important impact on the distribution of the number of NAT institutions in China.

**Fig. 7:**
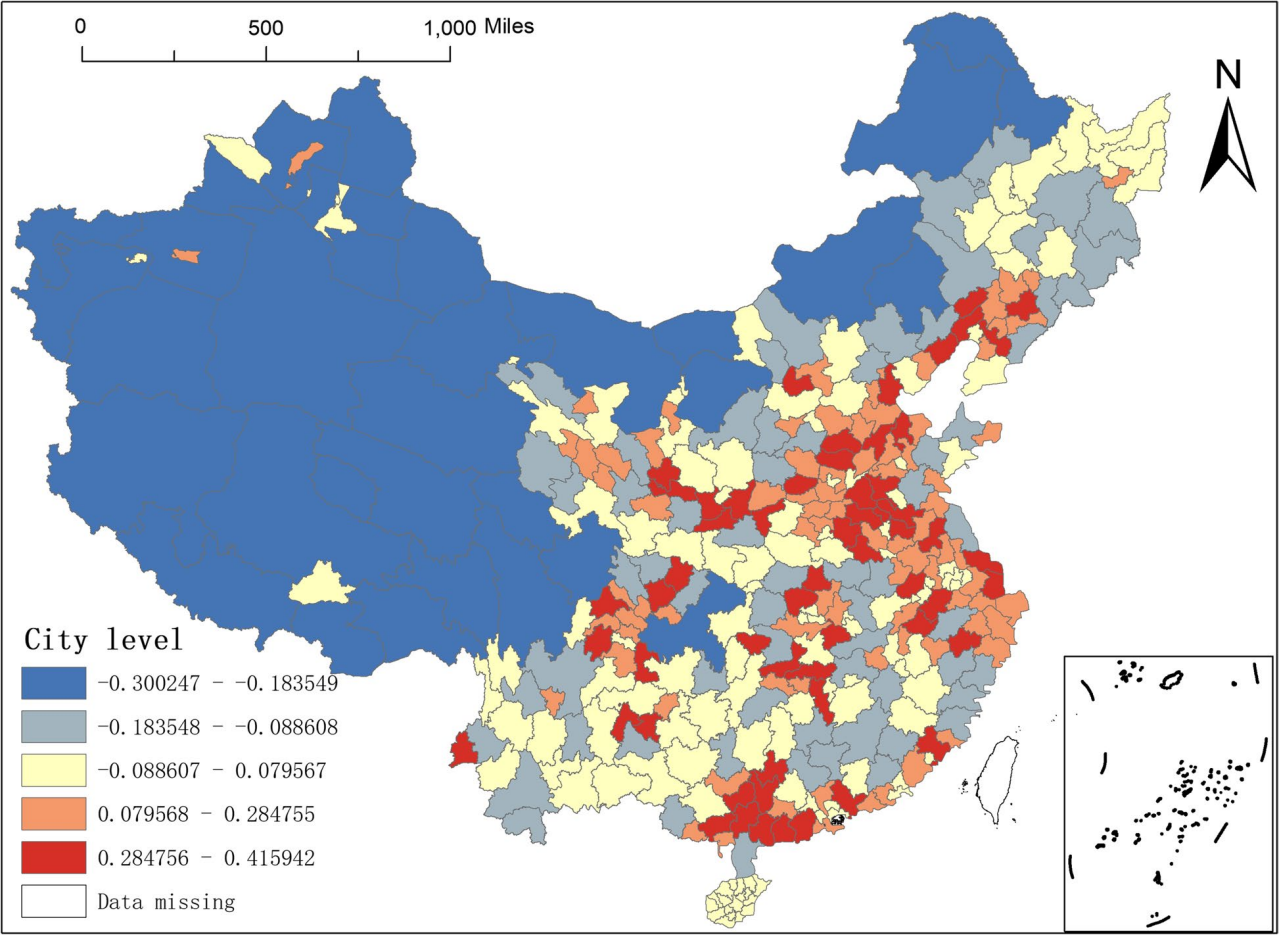
Spatial distribution of city level regression coefficients

Third, the regression coefficients of city level and altitude. In this paper, the city-level study units are classified into two types, namely, prefecture-level units and above prefecture-level units. Among them, above-prefecture-level units include Municipalities directly under the Central Government, provincial capital cities, and cities separately designated in the state plan. Based on the empirical analysis, it can be judged that as the transportation hubs and economic centers of each province and city, the prefecture-level units and above must strictly control the spread of the epidemic and must set up enough NAT institutions to deal with the risk of epidemic spread caused by the large-scale movement of people, namely, the higher the city level, the more NAT institutions in that place, and the statistical results in Fig. 7 confirm this judgment. The city-level regression coefficients of Shanghai, Tianjin, Xi'an, Chengdu, and Changsha, the provincial capitals, are all in the range of 0.2848–0.4159, which are in the distribution zone of regions with larger impact coefficients. However, the city-level regression coefficients of the northwest and southwest borderlands are negative.

**Fig. 8:**
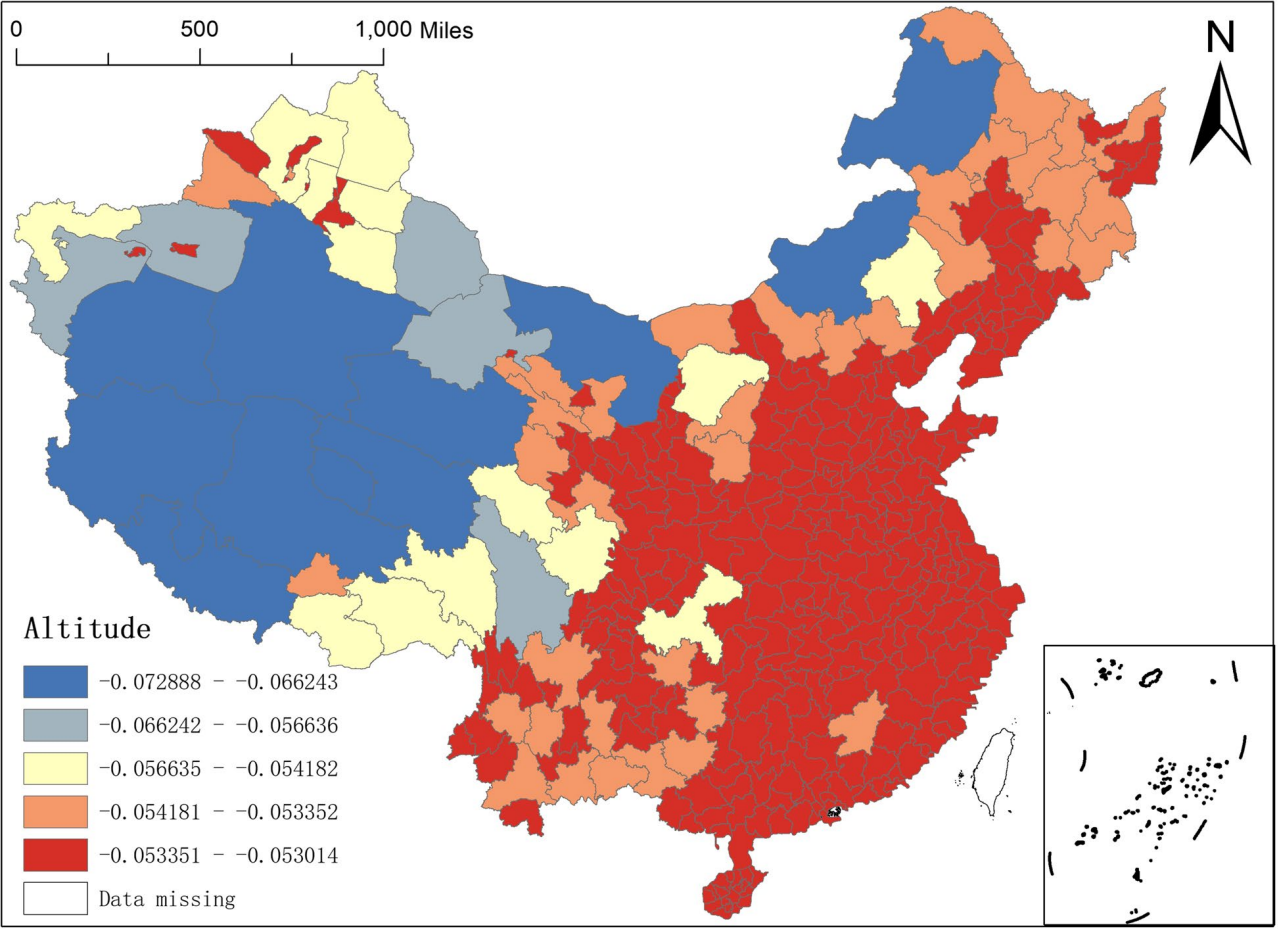
Spatial distribution of altitude regression coefficients

Altitude is the vertical distance of the ground above sea level. Generally speaking, the higher the altitude, the thinner the air is and the less suitable for human habitation and living. The negative altitude regression coefficient indicates that the higher the altitude, the fewer NAT institutions distributed, which shows consistency with empirical common sense. The spatial distribution of altitude regression coefficients in Fig. 8 clearly shows a northwest-southeast spatial difference. Specifically, altitude regression coefficients in the northwestern region range from -0.0729—-0.0566, with a greater degree of negative influence, while the altitude regression coefficients in the southeastern region range from -0.0542—-0.0530, with a smaller degree of negative influence.

In summary, the city level variable as a historical factor and the altitude variable as a geographical factor have some influence on the spatial distribution of NAT institutions in China, but also to a small extent, especially the altitude, which has a small absolute value of the regression coefficient. Therefore, there are other variables that have crucial effects on the distribution of NAT institutions.

**Fig. 9:**
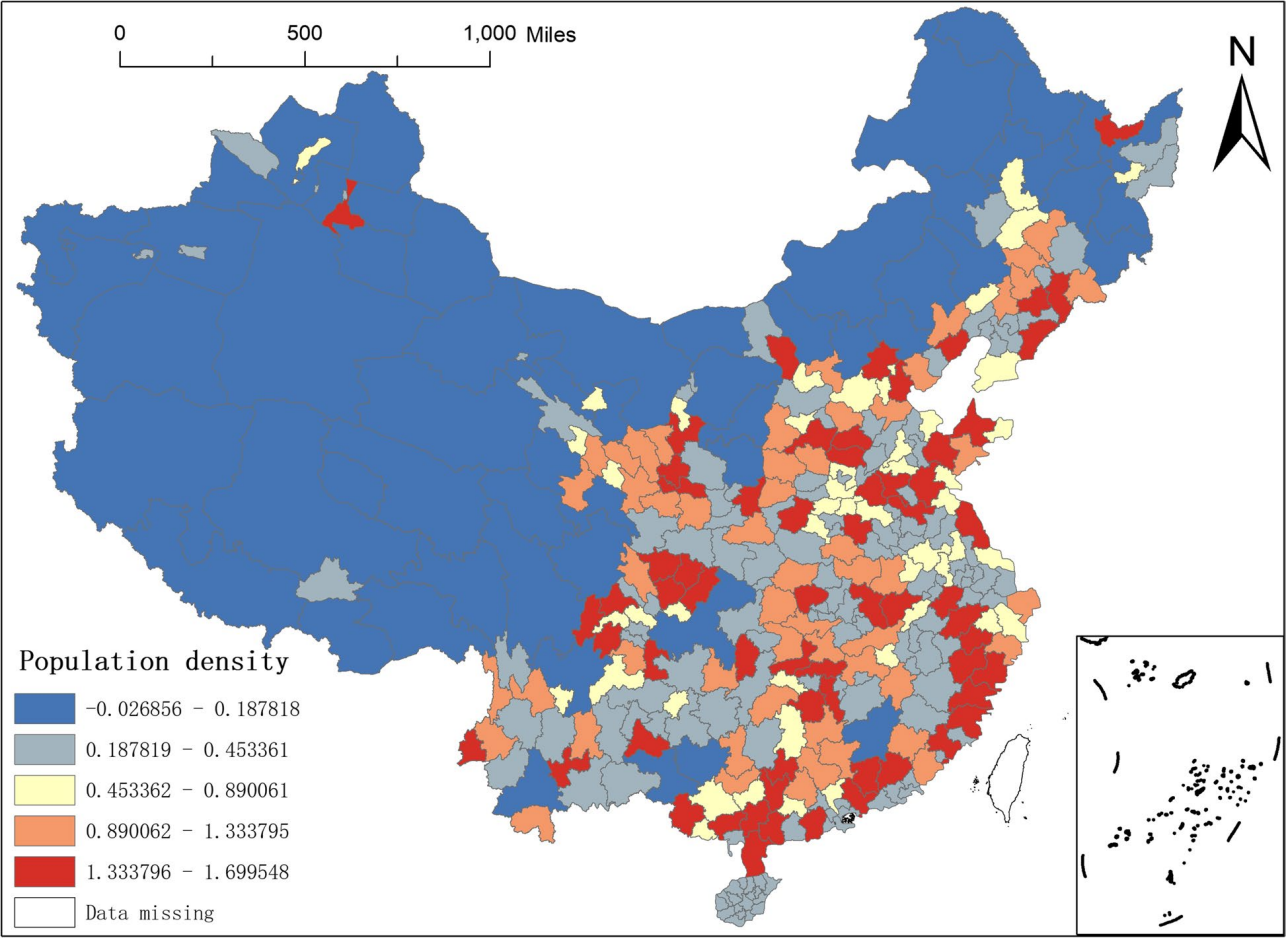
Spatial distribution of regression coefficients of population density in MGWR-SAR model

Fourth, the regression coefficient of population density. Population density is the number of people per unit land area, which is an important factor to examine to measure the economic development of a region and an important indicator of environmental pressure in a region. Therefore, this paper incorporates population density into the analytical framework. In Fig. 9, we can see that the minimum value of the regression coefficient of population density is -0.0269 and the maximum value is 1.6995. The regression coefficient of population density in the whole western region and most of the northeastern and southwestern cities is in the range of -0.0269–0.1878, and the absolute value of the regression coefficient is small, indicating that the population density has little influence on the distribution of NAT institutions in these regions. And the regression coefficient in the range of 1.3338–1.6995 is scattered in the central and eastern regions, such as Hebei, Shandong, Zhejiang, Sichuan, Guangdong, Guangxi, and Hunan, where the regression coefficients of population density are high, indicating that the population density in these regions has a greater impact on the distribution of NAT institutions.

**Fig. 10:**
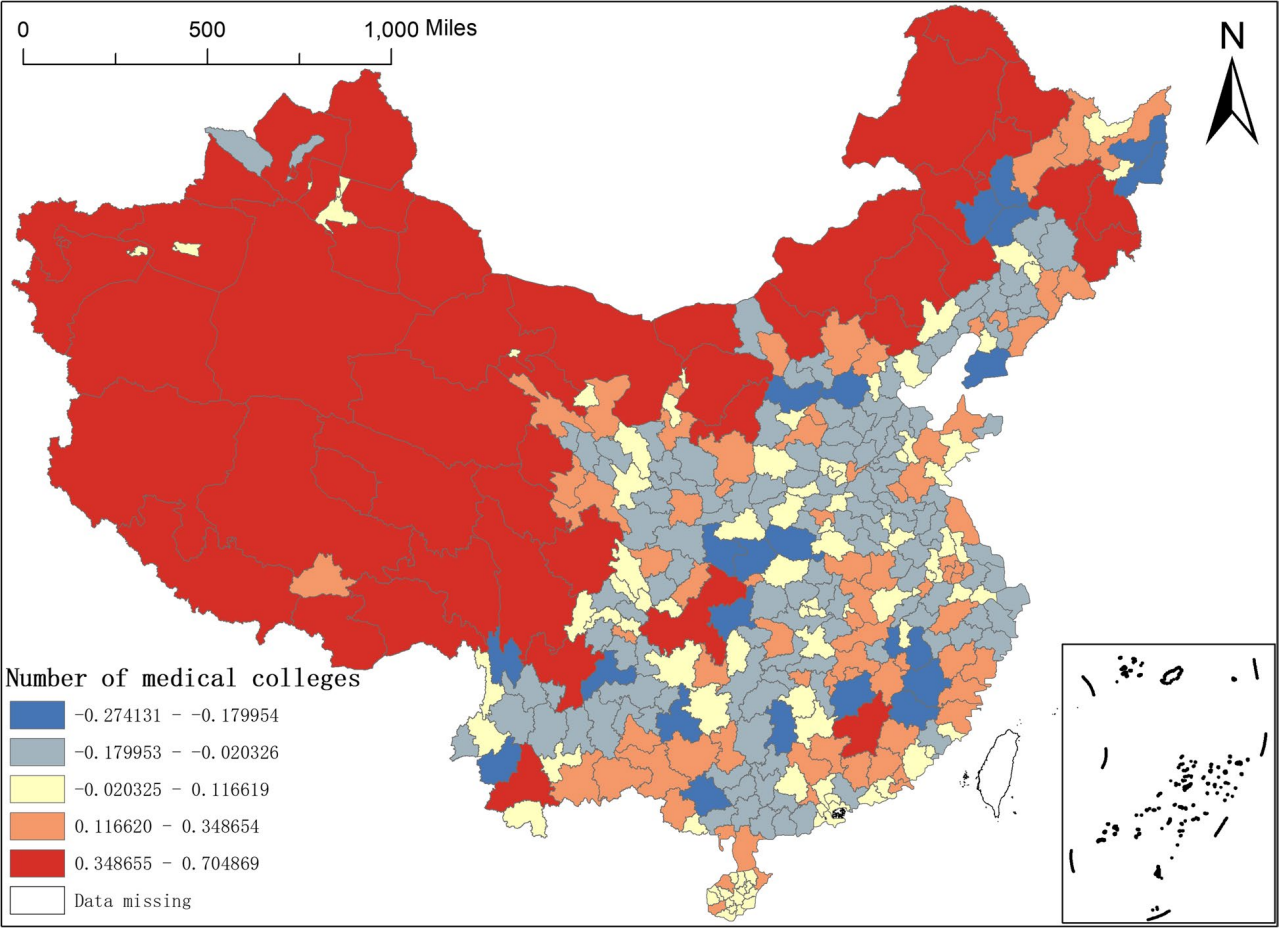
Spatial distribution of regression coefficients of the number of medical colleges

Fifth, the regression coefficient of the number of medical colleges and the number of tertiary hospitals. The number of medical colleges owned is the core variable characterising medical education in the region, and there are few studies in the current literature that addresses the imbalance in the allocation of quality medical resources in China from the perspective of medical education and other issues. In Fig. 10, the spatial coefficient distribution of the number of medical schools reflects a clear east–west difference. The regression coefficient values for the number of medical schools in the vast majority of the northwest and southwest regions, as well as Ganzhou in Jiangxi and Harbin, Mudanjiang and Yanbian Korean Autonomous Prefecture in the northeast, are largely within the larger range of 0.3487–0.7049, suggesting that the number of medical colleges has a greater impact on the distribution of NAT institutions in these regions, while the impact on the distribution of NAT institutions in the east is relatively small.

**Fig. 11:**
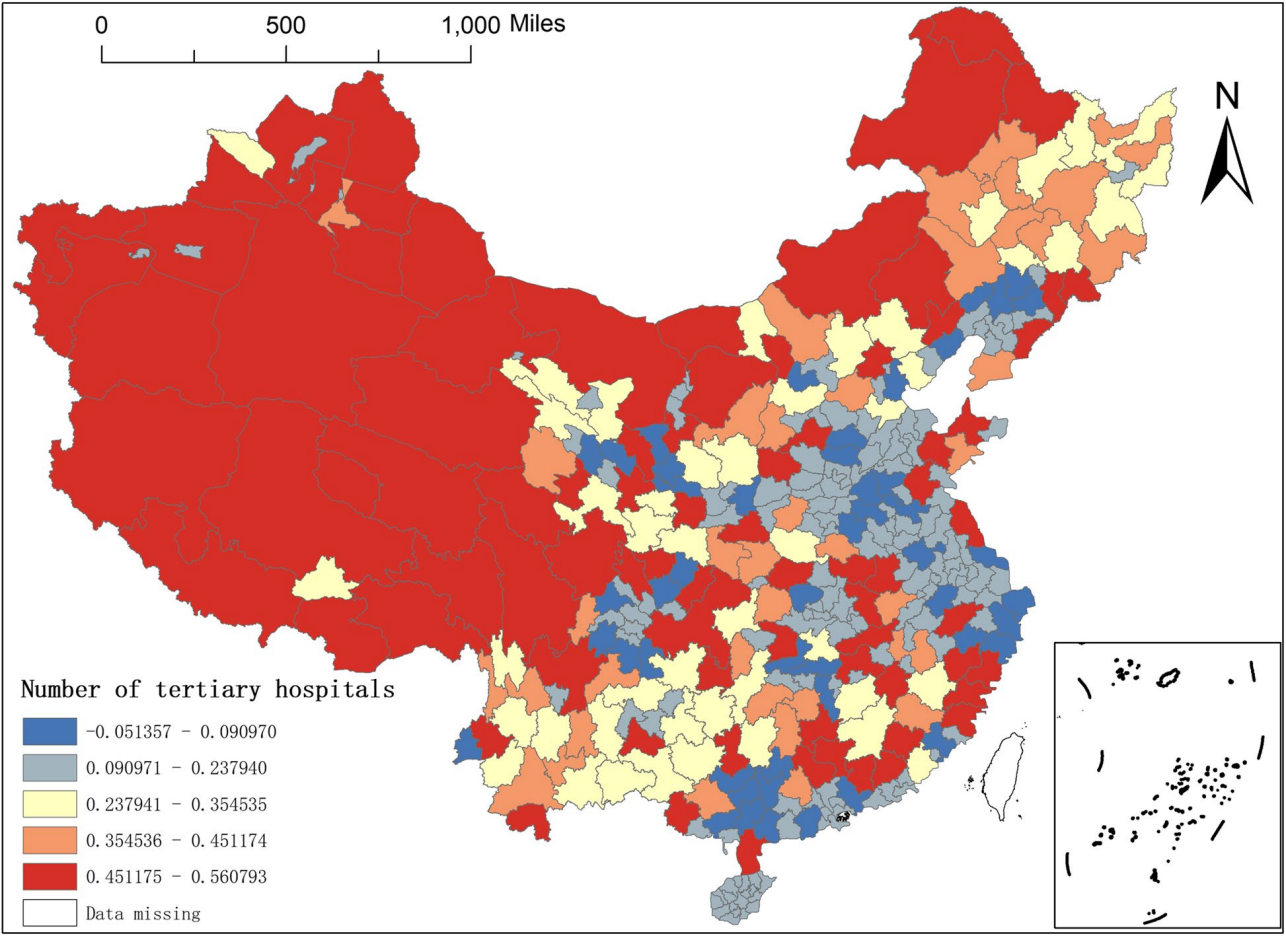
Spatial distribution of regression coefficients of the number of tertiary hospitals

Tertiary hospitals mainly refer to the large municipal hospitals directly under the national, provincial and municipal governments and the affiliated hospitals of medical colleges and universities—the number of tertiary hospitals is the core variable to characterise the local medical level. By the end of 2019, there were 1139 tertiary hospitals in China, mainly in the eastern region. Among the 361 prefecture-level municipal units, 30 cities have ten or more tertiary hospitals. Beijing, Shanghai, Guangzhou, Tianjin, Wuhan, Shenyang and Xi'an have more than 20 tertiary hospitals. They have 42, 36, 31, 29, 25, 21 and 20 tertiary hospitals, respectively, which usually also tend to be the highest quality local healthcare institutions, which can explain the number of tertiary hospitals as one of the main influencing factors on the distribution of NAT institutions in China. Still, differences in the number of tertiary hospitals between regions lead to spatial characteristics in their influence coefficients on the distribution of NAT institutions. The minimum value of the regression coefficient for the number of tertiary hospitals is -0.0514. The maximum value is 0.5608, which shows a significant spatial difference in the degree of influence of the number of tertiary hospitals on the distribution of NAT institutions in different regions. From the spatial distribution of the regression coefficients (Fig. 10), the areas with more substantial effects are mainly distributed in the western region, Changde in Hunan, Xiangyang and Huanggang in Hubei and the central region, as well as some cities in Shandong, Zhejiang, Fujian and Guangdong in the eastern region, etc. In particular, the maximum value of the influence coefficient appears in Jiuquan, Gansu Province; areas with weaker effects are mainly distributed in the eastern regions of Liaoning, Hebei, Jiangsu, Zhejiang, Guangdong, etc., in the east and Gansu and Sichuan in the west. At the same time, we compare the spatial distribution of the regression coefficients between the number of medical colleges and the number of tertiary hospitals (Figs. 10 and 11) and find one thing in common. The regression coefficients of these two variables have a relatively large explanation for the western region, indicating that the number of medical colleges and the number of tertiary hospitals have a rather significant impact on the distribution of NAT institutions in these areas.

**Fig. 12:**
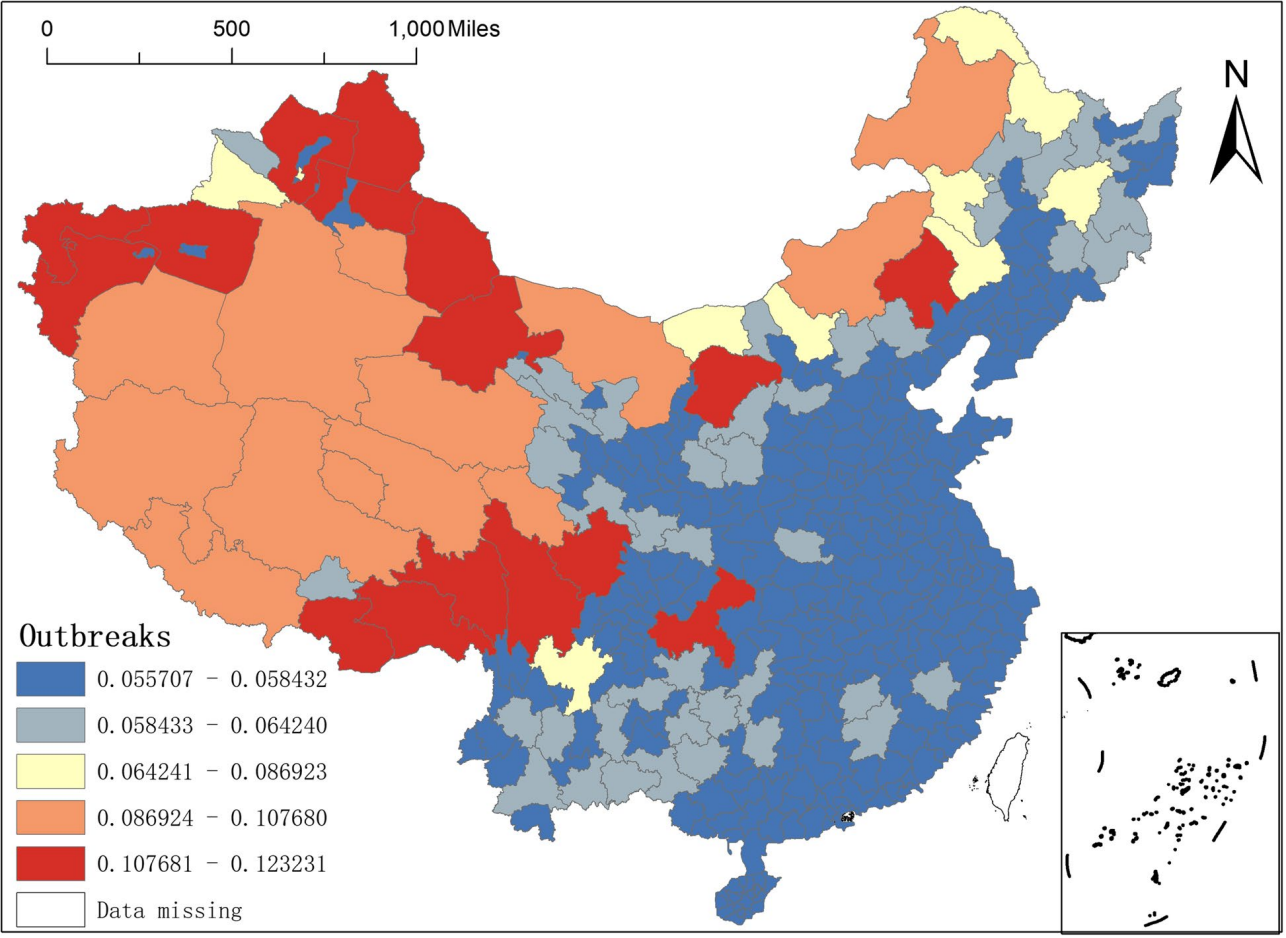
Spatial distribution of regression coefficients coefficients for the number of outbreaks case counts

Finally, there is the relationship between the number of outbreaks and the regression coefficient of the number of confirmed case counts of the COVID-19. Since the outbreak of the COVID-19 in late 2019 and early 2020, although the overall prevention and control effect in China has been effective, with no overall large-scale second outbreak. However, local areas have had two or more repeated outbreaks during this period, especially in areas near the border or near the sea where secondary outbreaks are more likely to occur, such as the city of Ruili in Dehong Prefecture, Yunnan Province, Manzhouli in Hulunbeier, Inner Mongolia, the Dalian region in Liaoning and areas of mass movement of people such as Shanghai and Beijing. And these areas should do more to prevent and control the outbreak, and need to set up a large number of NAT institutions to detect the virus. From the spatial distribution of the regression coefficient of the number of outbreaks (Fig. 12), the spatial distribution of the regression coefficient of the number of outbreaks shows a west–east distribution pattern. Specifically, the regression coefficient of the western region is more significant, basically in the range of 0.0869–0.1232, especially in Chongqing, Chifeng and Ordos in Inner Mongolia, Aba Tibetan and Qiang Autonomous Prefecture and Ganzi Tibetan Autonomous Prefecture in Sichuan, and Jiuquan in Tibet. In particular, the maximum value of the regression coefficient of the number of outbreaks is in the Altai region of Xinjiang; in the eastern part, the regression coefficient is minor, ranging from 0.0557 to 0.0642, and in particular, the minimum value of the regression coefficient of the number of outbreaks is in the city of Shihezi in Xinjiang.

**Fig. 13:**
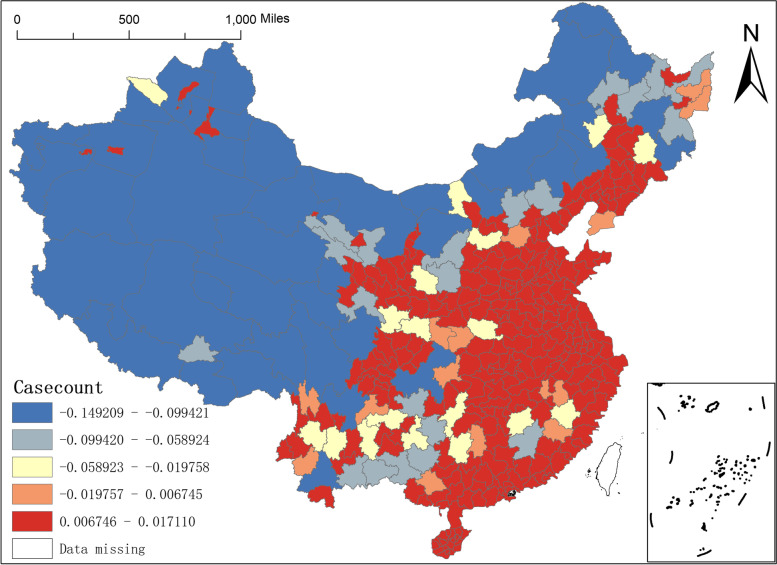
Spatial distribution of regression for the number of confirmed

The number of confirmed case counts of COVID-19 reflects the development of the epidemic in a region. It can be used to place higher demands on the prevention and control of the outbreak in each area, with few confirmed cases continuing to maintain and those with more confirmed cases learning from their mistakes to minimise the costs of outbreaks. Figure 13 reflects that the regression coefficients for confirmed cases have different effects on the distribution of NAT institutions, with a negative impact on the western region and a positive impact on the eastern part. Comparing Fig. 11 with Fig. 12, we can see that the spatial distribution of the regression coefficients for the number of confirmed case counts and the number of outbreaks of NAT for COVID-19 is precisely the opposite. The number of outbreaks had a robust positive effect on the distribution of NAT institutions in the western region. But the number of confirmed case counts had a strong negative impact on the distribution of NAT institutions in the west area. On the other hand, the number of outbreaks explained the distribution of NAT institutions less firmly in the eastern region. Still, the number of confirmed case counts explained the distribution of NAT institutions more strongly in the eastern area.

## Discussion

This article describes the spatial pattern of China's NAT institutions and their influencing factors on the scale of China's prefecture-level units. We have drawn a map of the spatial layout and spatial heterogeneity distribution of China's NAT institutions. ESDA is used to identify the degree of spatial aggregation of Chinese NAT institutions. Models such as OLS, OLS-SAR, GWR, GWR-SAR, MGWR, and MGWR-SAR are used to identify the determinants of Chinese NAT institutions spatial differences and spatial heterogeneity. Our paper has two main research findings. First, there are significant differences in the spatial allocation of NAT institutions in China, and spatial agglomeration characteristics are apparent. A comparative analysis reveals a gradual increase in the distribution of NAT institutions from west to east and that the projection points of institutions are denser in the eastern belt. For example, the city with the highest number of NAT institutions in Beijing, with 270 institutions, while Tibet has the lowest number of NAT institutions, with only 17. We use Moran's I to identify the degree of spatial aggregation of NAT institutions in China. The results of Moran's I values are all positive, indicating that the distribution of NAT institutions in China shows significant spatial distribution agglomeration. Secondly, there is substantial spatial heterogeneity in Chinese NAT institutions. After comparing the results of the OLS, OLS-SAR, GWR, GWR–SAR, MGWR and MGWR-SAR models, we find that the R^2^ of the MGWR-SAR model is 0.832, which is the maximum among all the models, indicating that the MGWR-SAR model has a better fit. This paper explores the spatial heterogeneity of NAT institutions in China based on the MGWR-SAR model. We find that variables such as city-level, population density, number of tertiary hospitals and number of outbreaks are important factors influencing the spatial heterogeneity of NAT institutions in China.

In addition, our study also has its limitations. The first limitation is that this paper uses the number of NAT institutions as a core indicator of NAT capacity, which is intuitive but does not consider factors such as NAT institutions size and testing capacity. The second limitation is that the paper analyses NAT as a whole rather than by category. NAT institutions in China are divided into three main categories: hospital-based, CDC-based, and third-party independent laboratories. And the spatial distribution of these three categories differs significantly. Hospital-based NAT institutions are the prominent NAT institutions. Their distribution is related to the population and medical market, following "less in the west and more in the east". The distribution in the eastern region is basically in a patchwork. There is also a pattern of "provincial capitals having more" hospital testing institutions, with cities such as the southeast coast having a relatively large number of hospital testing institutions. The number of third-party NAT institutions is relatively small and mostly located in the eastern region. At the same time, it is greatly influenced by the city level and is more often found in the capital cities of each province. The CDC type of NAT institution is set up by administrative jurisdiction, and their spatial distribution pattern differs significantly from that of hospital type testing institutions. Its most apparent distribution feature is that it is influenced by border areas, where more CDC testing institutions are set up, and the CDCs in border areas take on more tasks of NAT. In the future, it will be important to analyse and study the critical direction of China's epidemic management capacity for the above three types of NAT institutions classification.

## Conclusions

Unequal access to infectious disease testing for people in different regions can cause significant delays in all aspects of emerging outbreaks from initial detection to ultimate containment, leading to increasing morbidity and mortality and threatening global public health security.

Our study focuses on exploring the spatial characteristics of the NAT institutions and its determinants, especially on the question of whether it has spatial heterogeneity and spatial autocorrelation is discussed in depth. To this end, we take the OLS model for identifying the determinants of NAT institutions as a starting point, and also examine the OLS-SAR model that incorporates autoregressive terms (lagged terms), the GWR and MGWR models that can test for spatial heterogeneity, and the GWR-SAR and MGWR-SAR models that can examine both spatial heterogeneity and spatial autocorrelation. By comparing the goodness-of-fit metrics from multiple perspectives, we find that the MGWR-SAR model has a higher fitness to our research content.

We put forward some targeted policy recommendations. The government should allocate health resources rationally, optimise the spatial layout of nucleic acid testing facilities, and improve the capacity of each region to manage public health emergencies. At the same time, third-party testing facilities, as a powerful filler for public hospitals and CDCs, need to focus on their role in the public health emergency response system as a market force to alleviate the inequitable allocation of health resources between regions, as the number of public hospitals and CDCs cannot continue to increase. The construction of China's public health emergency response system should be internalized in the construction of the basic medical service system, including the reserve of personnel, equipment and technology, in order to prepare for possible future public health emergencies so that a public health emergency response plan can be activated in the first instance.

Finally, future examination of laboratory testing capabilities for emergency response to health emergencies in China should start from differentiating the functions of different types of institutions. 

## Data Availability

The factors influencing the layout of NAT institutions include epidemiological factors, economic and social factors, political factors, historical factors (and physical geographical factors. The epidemic factors include the number of epidemic outbreaks and the cumulative case numbers of new coronary pneumonia infections, with data obtained from publicly available data on the Chinese government website. The indicators that characterise the level of economic development include Population density, GDP per capita, urbanisation rate and city level, which comes from the "2019 Statistical Bulletin of Economic and Social Development" of each prefecture-level unit. The indicators of historical factors mainly include medical education, that is, the number of medical universities or medical colleges, the data of which comes from the directory of China Medical University (Medical College) and the affiliated hospitals. The data is obtained from the list of municipalities directly under the Central Government, provincial capital cities, and cities under separate planning. Please refer to Table 1 for the definition and source of all the above variables.
